# Synthesis and Antiviral Activity of Nanowire Polymers Activated with Ag, Zn, and Cu Nanoclusters

**DOI:** 10.3390/pharmaceutics17070887

**Published:** 2025-07-06

**Authors:** Thomas Thomberg, Hanna Bulgarin, Andres Lust, Jaak Nerut, Tavo Romann, Enn Lust

**Affiliations:** 1 Institute of Chemistry, University of Tartu, Ravila 14a, 50411 Tartu, Estonia; 2 Institute of Pharmacy, University of Tartu, Nooruse 1, 50411 Tartu, Estonia

**Keywords:** electrospinning, poly(vinylidene difluoride), metal nanoclusters, filtration efficiency, virucidal activity, H1N1, *SARS-CoV-2*

## Abstract

**Background/Objectives:** Airborne viral diseases pose a health risk, due to which there is a growing interest in developing filter materials capable of capturing fine particles containing virions from the air and that also have a virucidal effect. Nanofiber membranes made of poly(vinylidene fluoride) dissolved in N,N-dimethylacetamide and functionalized with copper, silver, and zinc nanoclusters were fabricated via electrospinning. This study aims to evaluate and compare the virucidal effects of nanofibers functionalized with metal nanoclusters against the human *influenza A virus* A/WSN/1933 (H1N1) and *SARS-CoV-2*. **Methods:** A comprehensive characterization of materials, including X-ray diffraction, scanning electron microscopy, microwave plasma atomic emission spectroscopy, thermogravimetric analysis, contact angle measurements, nitrogen sorption analysis, mercury intrusion porosimetry, filtration efficiency, and virucidal tests, was used to understand the interdependence of the materials’ physical characteristics and biological effects, as well as to determine their suitability for application as antiviral materials in air filtration systems. **Results:** All the filter materials tested demonstrated very high particle filtration efficiency (≥98.0%). The material embedded with copper nanoclusters showed strong virucidal efficacy against the *SARS-CoV-2* alpha variant, achieving an approximately 1000-fold reduction in infectious virions within 12 h. The fibrous nanowire polymer functionalized with zinc nanoclusters was the most effective material against the human *influenza A virus* strain A/WSN/1933 (H1N1). **Conclusions:** The materials with Cu nanoclusters can be used with high efficiency to passivate and kill the *SARS-CoV-2* alpha variant virions, and Zn nanoclusters modified activated porous membranes for killing human *influenza A virus* A7WSN/1933 (H1N1) virions.

## 1. Introduction

Airborne contaminants, including particulate matter and bioaerosols, pose a worldwide health risk and have been associated with the spread of various infectious diseases, including SARS, influenza, and COVID-19 [1,2,3]. Maintaining good air quality in settings like hospitals, homes, and industrial facilities has become critically important. *SARS-CoV-2* can spread from person to person via both direct and indirect transmission routes, particularly in crowded indoor spaces with poor ventilation [4,5,6]. The use of face masks has proven effective in significantly lowering viral transmission rates. Therefore, developing more effective air-cleaning filtering systems and face masks is essential. According to ISO 29463:2017 (High Efficiency Filters and Filter Media for Removing Particles from Air—Part 1: Classification, Performance, Testing and Marking) [7], high-efficiency air filters can remove more than 99.95% of particles at the most penetrating particle size [8,9]. The National Institute for Occupational Safety and Health (NIOSH) advises using N95 and N99 filters, which are capable of capturing approximately 300 nm aerosol particles with respective efficiencies of 95% and 99% [10,11]. Atmospheric aerosols are typically classified by size into those below and above 100 nanometers, while traditional non-woven microfiber filters are ineffective at capturing particles smaller than 300 nanometers. Therefore, special filter materials must be developed to capture smaller particles.

The COVID-19 pandemic has highlighted the critical importance of effective personal protective equipment, proactive air quality management, and filtering materials that can mitigate the spread of viral diseases [12,13,14]. The widespread use of PPE masks and other disposable devices has raised new environmental concerns [2,3,15]. Masks typically consist of multiple layers, each serving a specific function, such as a virucidal layer, a fluid barrier, and a particulate filtration layer [16,17,18], and they need several steps for production [18,19,20]. Disposable face masks have a restricted period during which they remain effective. In 2020, the monthly global consumption of face masks reached about 129 billion [21,22,23]. Therefore, developing filter materials with optimized porosity characteristics and advanced virucidal killing/passivating properties has great potential to overcome the limitations of traditional non-woven microfiber filters [24,25,26]. These advancements could help limit the spread of viruses and reduce the environmental impact of discarded masks [27,28,29].

The electrospinning method has been widely applied since 2007 for developing nanowire polymer separator materials for electrical double-layer capacitors (EDLCs) and Na-ion batteries [30,31,32]. Research indicates that the rate of solvent and ion adsorption (charging/discharging time) is significantly influenced by the hierarchical porosity, pore size distribution, and the ratio of micro-, meso-, and macropores in the separator materials [30,31,32]. Furthermore, the properties of the membranes depend on the electrospinning conditions (strength of the electric field, viscosity, and feed rate of the polymer solution), which affect the porosity characteristics of the resulting membranes [30,31,32]. The influence of separator material properties on the performance of energy storage systems is not studied systematically, as the energy stored in such systems is primarily determined by the properties of the active materials used, and commercially available separator materials are typically employed. However, the separator material and its compatibility with the electrolyte directly affect the system’s power capability—that is, how quickly energy can be stored and released, and our study clearly demonstrated this relationship [32,33,34]. The same electrospinning method can also be applied to the development of filter materials. The dependence of fibrous materials’ properties on electrospinning parameters has been thoroughly described elsewhere [35].

Different materials with distinct pore structures and filtering capabilities for capturing different sizes of aerosol particles can be created by varying electrospinning process parameters, such as environmental conditions (temperature and relative humidity), concentration and viscosity, and environmental conditions (temperature and relative humidity) [26,36,37]. The addition of various metals and their compounds, like silver [38,39,40], copper [41,42,43], zinc [36,44], and other metals (iron, tin, cobalt, titanium, etc.) and compounds [45,46,47], have been used to activate nanowire polymers to achieve virucidal activity.

This study primarily aims to evaluate and compare the antiviral effects of nanofibers functionalized with silver, copper, and zinc nanoclusters against both the human *influenza A virus* (*IAV*) A/WSN/1933 (H1N1) and *SARS-CoV-2* under the same experimental conditions. In this work, a comprehensive characterization of complex materials activated with Ag, Zn, and Cu nanoparticles was performed using various techniques, including X-ray diffraction, scanning electron microscopy, microwave plasma atomic emission spectroscopy, thermogravimetric analysis, contact angle measurements, nitrogen sorption analysis, mercury intrusion porosimetry, filtration efficiency, and virucidal tests [48,49,50]. This work employs various methods for the comprehensive physical characterization of the materials to understand the interdependence of the materials’ physical characteristics and biological effects. Furthermore, the characteristics mentioned above have been studied to determine if the given material is suitable for application as an antiviral material in an air filtration system, as the porosity and filtration performance are crucial factors of such materials.

## 2. Materials and Methods

### 2.1. Materials Used

Poly(vinylidene fluoride) (PVDF, average molecular weight ~ 275,000 g/mol, Sigma-Aldrich, St. Luise, MO, USA) was used as the primary matrix-forming polymer. N,N-dimethylacetamide (DMA, anhydrous, 99.8%, Sigma-Aldrich, St. Luise, USA) served as the solvent. Silver nitrate (AgNO_3_, ≥99.9%, Fischer, Loughborogh, UK), zinc chloride (ZnCl_2_, ≥98%, Fluka, Seelze, Germany), and copper(II) nitrate hydrate (Cu(NO_3_)_2_·2.5H_2_O, ≥99.99%, Sigma-Aldrich, St. Luise, USA) were employed as sources for activating nanoclusters without additional purification. A viscous solution was prepared by dissolving 28 wt% PVDF in DMA at 55 °C using a hot plate [24,25,26]. Solutions of AgNO_3_ and ZnCl_2_ were prepared in DMA with concentrations ranging from 0.25 wt% to 2.0 wt%, while Cu(NO_3_)_2_·2.5H_2_O solutions ranged from 0.25 wt% to 3.5 wt%. The mass percentages of the added salt are given relative to the prepared solution.

### 2.2. Electrospinning the Filter Materials

Nanofiber filter materials were fabricated using the EC-DIG electrospinning system equipped with climate control, manufactured by IME Technologies (Waalre, The Netherlands) (Figure S1 in Supporting Information). The general setup for the electrospinning experiments was consistent with our previous studies and is discussed in [26,32,34]. Solutions of PVDF in DMA (28 wt%), both with and without the addition of salts, were transferred into a plastic syringe (5 mL) equipped with a metallic needle with an inner diameter of 0.4 mm connected to a syringe pump (Harvard Apparatus, Holliston, MA, USA), which controlled the flow rate of the solution. The fibers were deposited onto aluminum foil positioned on a rotating collector with a diameter of 9 cm, spinning at 500 rpm. The distance of the needle tip to the rotating cylindrical collector was set to 9 cm, and the solution feed rate was set to 1 mL h^−1^. To ensure a uniform deposition, the needle tip was moved from side to side along a rail at a speed of 5 cm s^−1^, covering a total length of 15 cm. The electrospinning of the solutions was conducted at various constant voltages (from 9 to 21 kV) generated by a high-voltage DC system. All electrospinning experiments were performed at 23 ± 1 °C and a relative humidity of 60 ± 5%, with different parameters applied (Table S1 in Supporting Information).

### 2.3. Testing of Particle Filtration Efficiency and Breathability

A custom-designed atomizer was employed to produce a calibration aerosol consisting of 2 wt% NaCl and 3 μm polystyrene latex spheres (Lot No. 212,695 (net) 3 μm, Thermo Fisher Scientific, Waltham, MA, USA), as detailed in previous studies [24,25,26]. The aerosol flow was dried using a silica gel dryer, and its concentration was measured before and after passing through the test samples. Aerosol particles in the 5–500 nm size range were analyzed with a Fast Mobility Particle Spectrometer (FMPS, TSI Incorporated, Shoreview, MN, USA). In contrast, particles sized between 0.3 and 10 μm were measured using an Optical Particle Sizer (OPS, TSI Incorporated, Minnesota USA). An airflow velocity of 6 cm/s over a filtration area of 4.9 cm^2^ was ensured by a mass flow meter (TSI, model 4140) and regulated by a Bürkert 2/2-way solenoid valve (no. 00234303, Christian Bürkert GmbH, Ingelfingen, Germany). The background concentration of aerosol particles was measured for 3 min, and the average value was calculated. The filtering efficiency (*E*_eff_) was calculated as described in Refs. [24,25,26]. Testing was conducted on each filtering membrane material at a minimum of three distinct spots cut out from a single layer of material prepared by electrospinning.

The pressure drop measurement followed the EN 14683:2019 + AC:2019 Medical Face Masks—Requirements and Test Methods standard [51]; however, preconditioning at 85% relative humidity was not conducted. The test specimen was positioned between metal filter holders, and the pressure difference across the material was recorded. To verify leak tightness, the flow rate was measured before and after testing the material using a differential pressure manometer, CHY 886U, at an airflow rate of 8 dm^3^ min^−1^. Testing was conducted on each material at a minimum of three distinct spots.

### 2.4. Physical Characterization of Electrospun Filter Materials

A Zeiss EVO MA15 scanning electron microscope (SEM) equipped with an Oxford X-MAX 80 energy-dispersive X-ray (EDX) detector was used to study filter materials’ structure and morphology (SEM), elemental composition, and element distribution (SEM-EDX). A thin coating of platinum or carbon was applied to the surfaces of the materials before SEM-EDX analysis. Metal concentrations in the filter materials were determined using microwave plasma–atomic emission spectroscopy (MP-AES) (Agilent 4210 system, Santa Clara, CA, USA) [26,49]. ImageJ software version 1.53t was employed to analyze the fiber diameters and size distributions [50]. A two-tailed two-sample Student T-test, assuming equal variances, was used in order to evaluate the statistical significance of the difference in the fiber diameters of different materials. The alpha value was set at 0.05, and analysis was performed using Excel software (version 2505, Microsoft, Redmond, WA, USA).

Low-temperature nitrogen sorption measurements were performed at −195.8 °C using the ASAP 2020 system (Micromeritics, Norcross, GA, USA) to characterize the materials’ microporosity. The specific surface area (*S*_BET_) of the materials was calculated using the Brunauer–Emmett–Teller (BET) theory over a relative pressure (*p*/*p*_0_) range of 0.05 to 0.1 and the total pore volume (*V*_sum_) at *p*/*p*_0_ = 0.95 [52,53].

The meso-macroporous structure of the filters was characterized using mercury intrusion porosimetry (AutoPore IV (Micromeritics), ultrapure triple-distilled mercury with purity of 99.9995%), within the pressure range from 0.01 to 410 MPa [54,55,56]. Before measurements, the samples were degassed under a vacuum at 100 °C for one hour. The use of mercury porosimetry in addition to gas sorption measurements was necessary to gain insights into larger pores, i.e., mesopores and macropores, as gas sorption measurements provide information only about the microporosity of materials.

The thermal stability and purity of the electrospun materials were examined through thermogravimetric analysis (TGA) using a NETZSCH STA 449 F3 system (Selb, Germany) equipped with an Al_2_O_3_ sample holder. The analysis was conducted over a temperature range of 25 °C to 1000 °C, with a heating rate of 10 °C min^−1^ and a gas flow rate of 50 cm^3^ min^−1^ using nitrogen (99.999%, Linde plc, Dublin, Ireland) and synthetic air (20 vol% oxygen and 80 vol% nitrogen, 99.999%, Linde plc, Ireland), respectively [25,26,57].

X-ray diffraction (XRD) was used to analyze the crystallographic structure of the membranes. XRD measurements were carried out using a Bruker D8 diffractometer (Bruker Corporation, Billerica, MA, USA) at 25 ± 1 °C. The setup employed CuKα radiation and a LynxEye position-sensitive detector (Stockholm, Sweden), with a scanning step size of 0.01° and a count time of 2 s per step [58,59].

The preliminary hydrophilicity of the electrospun filter materials was assessed by measuring the contact angle using droplets of ultrapure water (Milli-Q+, Burlington, MA, USA, specific resistance 18.2 MΩ·cm) applied directly onto the filter surfaces [24,26,60].

### 2.5. Virus Strain and Virucidal Activity Determination

The virucidal activity measurement of fibers activated with Ag, Zn, or Cu nanoclusters, as well as unactivated PVDF fibers (control material), was conducted against the human *influenza A virus* (*IAV*) A/WSN/1933 (H1N1) strain following the ISO 21702:2019 (Measurement of Antiviral Activity on Plastics and Other Non-Porous Surfaces) standard [61], as well as a recombinant strain of severe acute respiratory syndrome coronavirus 2 (*SARS-CoV-2*) (ISO 21702:2019 standard, Measurement of Antiviral Activity on Plastics and Other Non-Porous Surfaces) [24,25,26].

Human *IAV* was cultured in confluent monolayers of Madin–Darby canine kidney (MDCK) cells (ATCC, Teddington, UK) maintained in Dulbecco’s Modified Eagle Medium (DMEM, Corning, Corning, NY, USA, #10-013-CV), supplemented with 0.2 wt% bovine serum albumin (BSA), TPCK-treated trypsin at a 1:1000 dilution (Sigma, #T1426), and 1% *v*/*v* Penicillin/Streptomycin solution (Gibco, Waltham, MA, USA, #15070-063), hereafter referred to as virus growth medium (VGM). A viral suspension with a concentration of 10^7^–10^8^ TCID_50_ mL^−1^ in VGM was used for experiments [25].

The *SARS-CoV-2* (Wuhan-Hu-1 strain) used in virucidal testing had the gene encoding the spike protein replaced by that of the alpha variant. For the visualization of infected cells, a mNeonGreen marker was attached to the C terminus of the ORF7a protein of the virus, designated as *SARS-CoV-2* NG [24,26,62]. *SARS-CoV-2* was propagated in VeroE6 cells (epithelial kidney cells from African green monkey, ATCC, United Kingdom) cultured in virus growth medium (VGM). For experiments, a stock solution of *SARS-CoV-2* NG with an approximate concentration of 7 × 10^7^ TCID_50_ units ml^−1^ was diluted tenfold in phosphate-buffered saline (PBS) [24,26,62].

To eliminate potential contamination, all samples were sterilized using UV-C radiation. Subsequently, 30 mm × 30 mm sections of the test or control materials were inoculated with 200 μL of the virus suspension (*IAV* or *SARS-CoV-2*) and covered with a 20 mm × 20 mm piece of PVC (poly(vinyl chloride) to achieve an even spread of the suspension. The samples were incubated from 0 to 12 h at 25 ± 2 °C, followed by washing with 10 mL of Soybean Casein Lecithin Polysorbate Broth to remove residual virus or 10 mL of VGM solution in the case of human *IAV* or *SARS-CoV-2*, respectively [24,26,62].

The concentration of infectious virus was determined using the TCID_50_ assay, with the Spearman–Karber algorithm applied for analysis [24,26]. Mean viral titers for each sample were determined from a minimum of three independent experiments. The reduction in viral concentration (Δlog c) was subsequently calculated as Δlog*c* = log(*c*_0h_) − log(*c_x_*_h_), where *c*_0h_ and *c_x_*_h_ represent the viral concentrations at initial contact (0 h) and after x hours of exposure, respectively (*x* = 1, 2, 4, 8, or 12 h).

## 3. Results and Discussion

### 3.1. Physical Characterization Results of Electrospun Filter Materials

**Figure 1:**
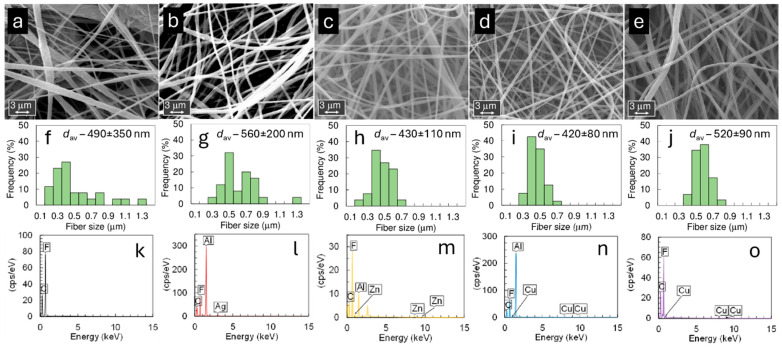
SEM images (**a**–**e**), fiber diameter distribution graphs (**f**–**j**), and SEM-EDX spectra (**k**–**o**) are presented for electrospun filter materials prepared from a 28 wt% PVDF + DMA solution at 9 kV (**a**,**f**,**k**), and with the addition of 2.0 wt% AgNO_3_ at 15 kV (**b**,**g**,**l**), 2.0 wt% ZnCl_2_ at 15 kV (**c**,**h**,**m**), 0.25 wt% Cu(NO_3_)_2_·2.5H_2_O at 17 kV (**d**,**i**,**n**), and 3.5 wt% Cu(NO_3_)_2_·2.5H_2_O at 17 kV (**e**,**j**,**o**). Scale bars are included on the SEM images (**a**–**e**).

The SEM-EDX data in Figure 1 are comparable to results reported in papers [59,63,64]. The fibers accumulate randomly on the collector surface, forming thin fibrous membranes that exhibit different visual densities. The visual densities are higher for material produced from 2.0 wt% ZnCl_2_ and 0.25 wt% Cu(NO_3_)_2_·2.5H_2_O solutions than for other membranes deposited at 15 kV. The thickness of the fibers depends on electric field strength, i.e., on applied voltage, deposition distance, and length, as well as on the concentration of PVDF, AgNO_3_, ZnCl_2_, and Cu(NO_3_)_2_·2.5H_2_O in DMA (as demonstrated in Table S1, Supporting Information) [24,26,37]. The strength of the electric field exerts a moderate influence on the structure of fibers—the fiber diameter of unmodified PVDF materials increases from 490 ± 340 nm to 600 ± 380 nm when the electric field strength increases from 1 to 1.44 kV cm^−1^ [25]. The distribution of carbon and fluoride in the PVDF mat is random and uniform, based on SEM-EDX data [24,25].

AgNO_3_, ZnCl_2_, and Cu(NO_3_)_2_⋅2.5H_2_O salts were added to a PVDF solution in DMA, achieving fixed concentrations ranging from 0.25 to 2.0 wt% (for Cu(NO_3_)_2_⋅2.5H_2_O up to 3.5 wt%) to achieve activated fibers with metal nanoparticles. Data in Figure 1 and Table S1 (Supporting Information) indicate that the fiber diameter increases from 470 ± 90 nm to 670 ± 260 nm with the increase in Cu(NO_3_)_2_·2.5H_2_O concentration in spinning solution from 0.25 wt% to 3.5 wt% at a voltage of 15 kV, and the differences in fiber diameters were statistically significant independent of the salt concentration used. The effect of AgNO_3_ concentration on fiber diameter was minimal when low amounts of salt were added (0.25 and 0.75 wt%). However, there was a slight increase in fiber diameter when 2.0 wt% of AgNO_3_ was added [25]. The differences between the fiber diameters were not statistically significant in the case of filter materials produced from spinning solutions containing AgNO_3_. The effect of ZnCl_2_ concentration is more pronounced—the average diameter systematically decreases from 710 ± 200 nm (0.25 wt% ZnCl_2_) to 430 ± 110 nm (2.0 wt% ZnCl_2_) at a voltage of 15 kV (Table S1, Supporting Information). The difference in the fiber diameter was not statistically significant in the case of materials produced from solutions containing 0.25 wt% ZnCl_2_ or 0.75 wt% ZnCl_2_. The fiber diameter of the material produced from solutions containing 2.0% ZnCl_2_ was statistically significantly smaller compared to materials produced with lower ZnCl_2_ content. The decrease in fiber diameter can be attributed to the enhanced conductivity of the electrospinning solution with increasing ZnCl_2_ concentration. At the same wt%, the ZnCl_2_ solution demonstrates higher conductivity than AgNO_3_. As the repulsion of charges on the surface of the electrospinning jet causes stretching, solutions with higher conductivity are expected to yield fibers displaying a finer morphology with fewer bead-like defects. Due to the higher viscosity of the solutions, it is anticipated that the smaller ionic radius of Zn^2+^ may impact the results. PVDF fibers modified with Zn or Ag nanoclusters, prepared at an electric field strength of 1.67 kV cm^−1^ and a solution feed rate of 1 mL h^−1^, exhibited very smooth nanofibers (Figure 1b,c). Still, unmodified PVDF fibers did exhibit some defects, like small beads (Figure 1a). It can be concluded from the obtained results that the addition of salt has a significant effect on the morphology and fibrous structure of fabricated filter materials. This is important because it influences the inter-fiber spacing, i.e., the porosity of the materials (discussed later), which, in turn, affects both the filtration efficiency and breathability of the filter materials—key factors determining their suitability for use in filtration applications. In general, as the fiber diameter increases, filtration efficiency increases because the inter-fiber space, i.e., the pore diameter, decreases, allowing smaller particles to be captured. However, breathability decreases in contrast to filtration efficiency, as materials with smaller fibers have a denser structure, which hinders airflow through the material [23,65,66].

The results of EDX analysis show that the amount of Ag, Cu, and Zn nanoparticles within the fibers is related to the salt concentration in the spinning solution. The EDX results also verified a uniform distribution of these nanoparticles throughout the electrospun filters (see Figure 1). These findings agree with MP-AES measurement results (Table S2, Supporting Information). During the electrospinning, the solvent evaporates, leaving only PVDF and metal nanoparticles in the fibers.

**Figure 2:**
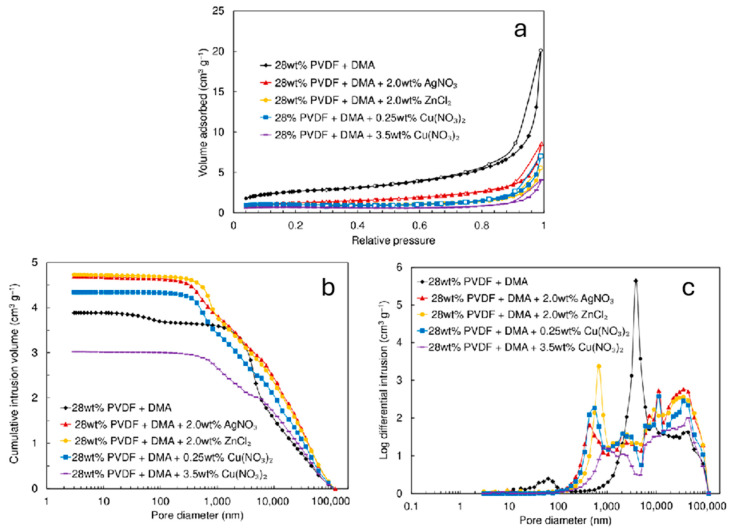
(**a**) Nitrogen sorption isotherms (adsorption—filled marks; desorption—open marks), (**b**) mercury intrusion curves, and (**c**) pore size distribution graphs derived from mercury porosimetry data for electrospun filter materials produced from a 28 wt% PVDF + DMA solution at 9 kV, as well as those with added 2.0 wt% AgNO_3_ at 15 kV, 2.0 wt% ZnCl_2_ at 15 kV, 0.25 wt% Cu(NO_3_)_2_·2.5H_2_O at 17 kV, and 3.5 wt% Cu(NO_3_)_2_·2.5H_2_O at 17 kV.

The form of the N_2_ sorption isotherms (Figure 2a) indicates that the materials examined exhibit low microporosity [52,53,54]. Furthermore, the mesoporosity of the membranes appears to be moderate, which is evidenced by the slight hysteresis noted between the adsorption and desorption curves of the N_2_ sorption isotherm (Figure 2a). However, the mesoporosity seems to be more expressed for materials without metal nanoclusters deposited into/onto membranes. Both the total pore volume (*V*_tot_) and the specific surface area (*S*_BET_) decrease as the concentration of Ag, Zn, and Cu nanoclusters increases (Figure 2a and Table 1). When the maximum amounts of Ag, Zn, and Cu are loaded onto the materials, the specific surface area decreases nearly twice compared to unmodified polymer material fibers.

The data from mercury intrusion porosimetry [54,55,56] (Figure 2b,c) indicate that the membranes possess a meso-macroporous structure. The predominant pores are mesopores, with diameters ranging from 30 to 200 nm. Also, some macropores with diameters between 300 and 400 μm are present. The introduction of Ag, Zn, and Cu nanoclusters only slightly reduces the total pore volume of the fiber materials (Figure 2b and Table 1). For fibers containing Cu nanoclusters, a significant decrease in intruded Hg volume is noted as the concentration of Cu(NO_3_)_2_⋅2.5H_2_O increases from 0.25 to 3.5 wt%. The highest Hg volume accumulated was recorded for 2.0 wt% AgNO_3_ and ZnCl_2_ materials with the highest *S*_BET_ and *V*_tot_ values. The Hg volume introduced into the nanocluster-modified membrane was slightly higher than that in pure PVDF fibers. This can be explained by the more homogeneous and less defective fibrous structure in the materials containing Ag, Zn, and Cu nanoclusters compared to the pure PVDF fibrous material, which exhibits many defects. Hg intrusion porosimetry data indicate that highly meso-macroporous PVDF nanofibrous material has been formed under fixed electrospinning conditions due to the formation of material with voids between the nanofibers [24,25,26].

The XRD results in Figure S2 (Supporting Information) reveal a significant broadening of the diffraction peaks for PVDF nanofibrous filter materials functionalized with Cu, Ag, and Zn nanoclusters. Pure PVDF exhibits a monoclinic structure (*γ*-PVDF), as reported by other authors elsewhere [59,64,67].

In contrast, the presence of Ag and Zn nanoclusters initiated the formation of an orthorhombic structure of PVDF (the beta *β*-PVDF form) (Figure S2, Supporting Information). The data presented in the literature [59,63,64] indicates that PDVF crystallizes in the *β* form when fillers such as BaTiO_3_, TiO_2_, or metallic nanoparticles are introduced. The XRD spectra did not show any reflections characteristic of AgNO_3_ or ZnCl_2_; thus, the salt decomposition occurred, and the resulting fibers did not contain salts and anions. Additionally, the nanoclusters within the nanofibers seem to be screened by a thin layer of PVDF. This is supported by the fact that there was no significant chemical dissolution of Zn, Ag, and Cu in 0.1 M HNO_3_ aqueous solution that could be detected with the MP-AES method. The study revealed that materials containing copper nanoclusters formed *β*-PVDF (Figure S2) [59,63,64], indicating that in these materials, copper is present not as Cu(0) and/or Cu(NO_3_)_2_⋅2.5 H_2_O but rather as “basic copper nitrate”, specifically Cu(NO_3_)(OH)_3_, which is also known as Rouaite [68].

Furthermore, EDX data in Figure 1 demonstrate that while signals from Ag and Zn are detectable, their intensities are significantly lower than those of fluorine and carbon, i.e., signals from PVDF polymer film. Al from the foil on the rotating drum used to collect the electrospun materials was also detected using EDX. The minor rise in metal concentration within the fibers is likely due to the limited surface activity of the supporting film, which can be attributed to the screening effect of the PVDF surface layer.

**Figure 3:**
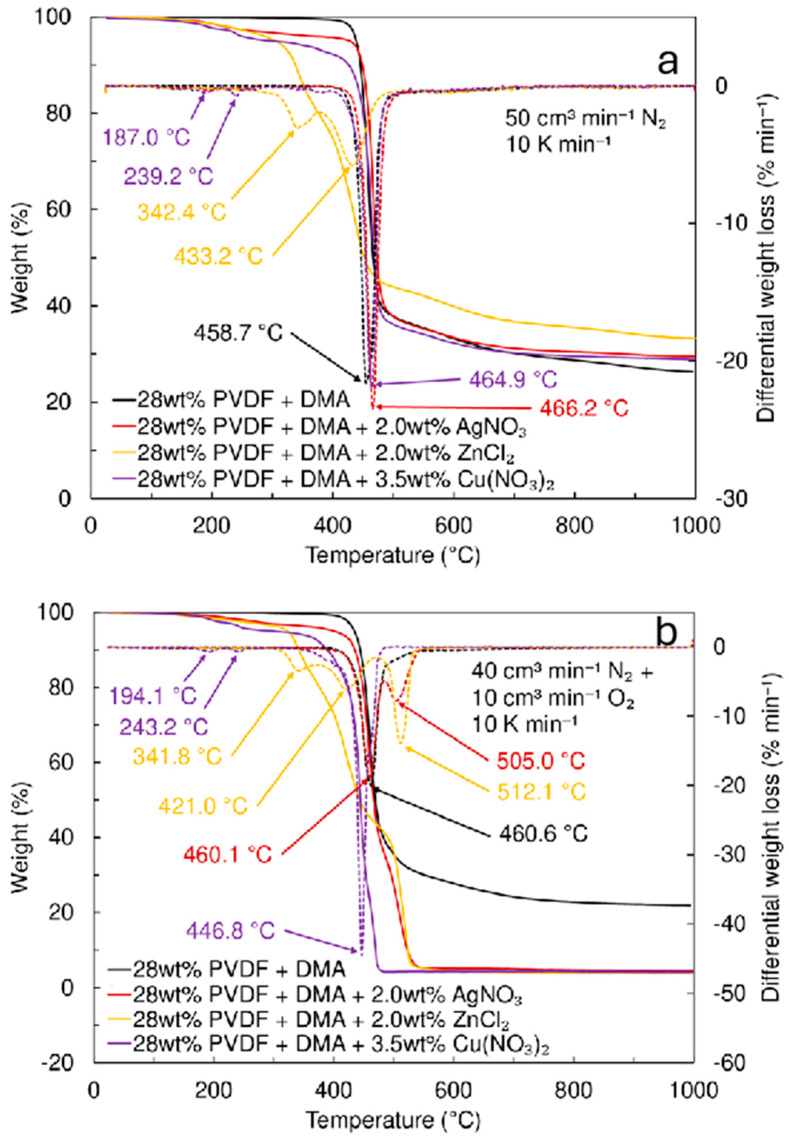
Thermogravimetric analysis (weight loss—solid line, differential weight loss—dotted lines) in (**a**) nitrogen and (**b**) synthetic air (mixture of 20 vol% oxygen and 80 vol% nitrogen) for different electrospun filter materials fabricated using the solutions of 28 wt% PVDF + DMA at voltage 9 kV, and with the addition of 2.0 wt% AgNO_3_ at 15 kV, 2.0 wt% ZnCl_2_ at 15 kV, and 3.5 wt% Cu(NO_3_)_2_·2.5H_2_O at 17 kV.

The electrospun materials were analyzed using the thermogravimetric method (Figure 3). The results indicate that a highly pure PVDF material was produced. Thermal decomposition began at 460.1 °C in a nitrogen (inert gas) atmosphere (Figure 3a) and at 460.6 °C in synthetic air (20 vol% oxygen and 80 vol% nitrogen) (Figure 3b). The nanofiber material modified with Ag nanoclusters demonstrated stability at temperatures similar to that of the pure PVDF membrane. At 505 °C, a small new peak was observed, marking the complete decomposition of PVDF [24,25,26]. The behavior of PVDF modified with Zn nanoclusters is more complex, displaying three distinct peaks (Figure 3b) and showing a gradual decomposition of PVDF occurring at 341.8 °C and 421 °C, with complete decomposition taking place at 512.1 °C. Interestingly, in a pure nitrogen atmosphere, the same peaks were observed as in synthetic air (Figure 3a); however, the third peak at higher temperatures, which was noted for pure PVDF and Ag nanocluster-modified materials, was absent. In both atmospheres, the main decomposition of the Cu-modified materials occurred at ∼460 °C (Figure 3). For materials containing Rouaite, two additional small peaks were observed at 190 °C and 240 °C, explained by the decomposition of Rouaite complex hydroxide and the desorption of OH^−^ from the Rouaite nanoclusters. The material synthesized from 28 wt% PVDF + DMA with 3.5 wt% Cu(NO_3_)_2_·2.5H_2_O showed a residual mass of approximately 4.2 wt%, compared to 22 wt% for pure PVDF. Thus, thermogravimetric data indicate that the nanofiber materials are thermally stable over a wide temperature range (up to 430–450 °C), making them suitable for various (even high-temperature) filtration applications.

**Figure 4:**
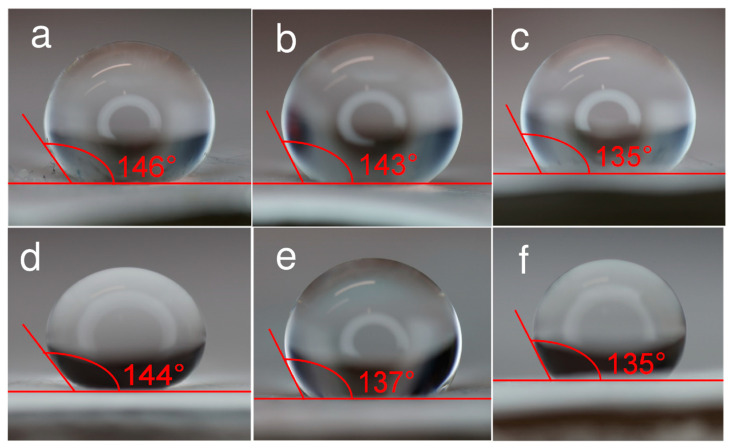
Microscopic images of wetting angle for different electrospun filter materials fabricated using a solution of (**a**) 28 wt% PVDF + DMA at voltage 9 kV, and with the addition of (**b**) 2.0 wt% AgNO_3_ at 15 kV, (**c**) 2.0 wt% ZnCl_2_ at 15 kV, (**d**) 0.25 wt% Cu(NO_3_)_2_·2.5H_2_O at 17 kV, (**e**) 2.0 wt% Cu(NO_3_)_2_·2.5H_2_O at 17 kV, and (**f**) 3.5 wt% Cu(NO_3_)_2_·2.5H_2_O at 17 kV.

The hydrophilicity of the surfaces [26,34,62] was measured with the contact angle method (Figure 4). A droplet of ultrapure water (10 μL) was carefully dripped onto the surfaces of different membranes. Three measurements for the same membrane material revealed minimal variation (±1°) in the contact angle values. The largest contact angle recorded was 146 ± 2° for the non-activated filter, making it the most hydrophobic of the filter materials tested. The contact angle values for different activated materials varied: 135 ± 2°, 137 ± 2°, and 144 ± 2° for filter materials with 3.5 wt%, 2.0 wt%, and 0.25 wt% Cu(NO_3_)_2_·2.5H_2_O (Figure 4d–f). The contact angles for membranes containing 2.0 wt% ZnCl_2_ and 2.0 wt% AgNO_3_ in the spinning solution were 135 ± 2° and 143 ± 2°. These results indicate that all surfaces measured were relatively hydrophobic, i.e., poorly wetting. However, the addition of metal nanoclusters makes fibers slightly more hydrophilic. This is particularly true when membranes are spun from solutions containing 2.0 wt% ZnCl_2_ and 3.5 wt% Cu(NO_3_)_2_·2.5H_2_O.

### 3.2. Particle Filtration Efficiency and Pressure Drop Values for Electrospun Filter Materials

The filtration efficiency (*E*_eff_) and breathability of the electrospun materials were evaluated using a custom-designed system, with the results summarized in Table 2. Filtration efficiency was measured for aerosol particles sized 100 nm, 300 nm, and 3000 nm [24,25,26]. All electrospun filter materials incorporating Ag, Zn, and Cu nanoclusters demonstrated exceptionally high filtration efficiency, reaching at least 98%. *E*_eff_ only somewhat increases with the thickness of the material. However, there is a nearly linear relationship between the pressure drop and the thickness of the material (Table 2).

The electrospun filter material prepared from pure PVDF achieved a higher filtration efficiency of 99.5% only for large particles with a diameter of 3000 nm. At the same time, there was a strong dependency between *E*_eff_ and filter material thickness for smaller particles (100 and 300 nm). The filtration efficiency *E*_eff_ increased significantly with material thickness (Table 2). This effect can be attributed to the different morphology and porosity characteristics of materials electrospun from pure PVDF solutions compared to those that contain metal nanoclusters (Figure 1 and Figure 2, Table 1 and Table 2). Including salts enhances the conductivity of the polymer solution, resulting in greater stretching of the solution jet during electrospinning. This promotes the formation of a more uniform fibrous structure with slightly increased hydrophilicity.

### 3.3. Results of Virucidal Activity Tests

**Figure 5:**
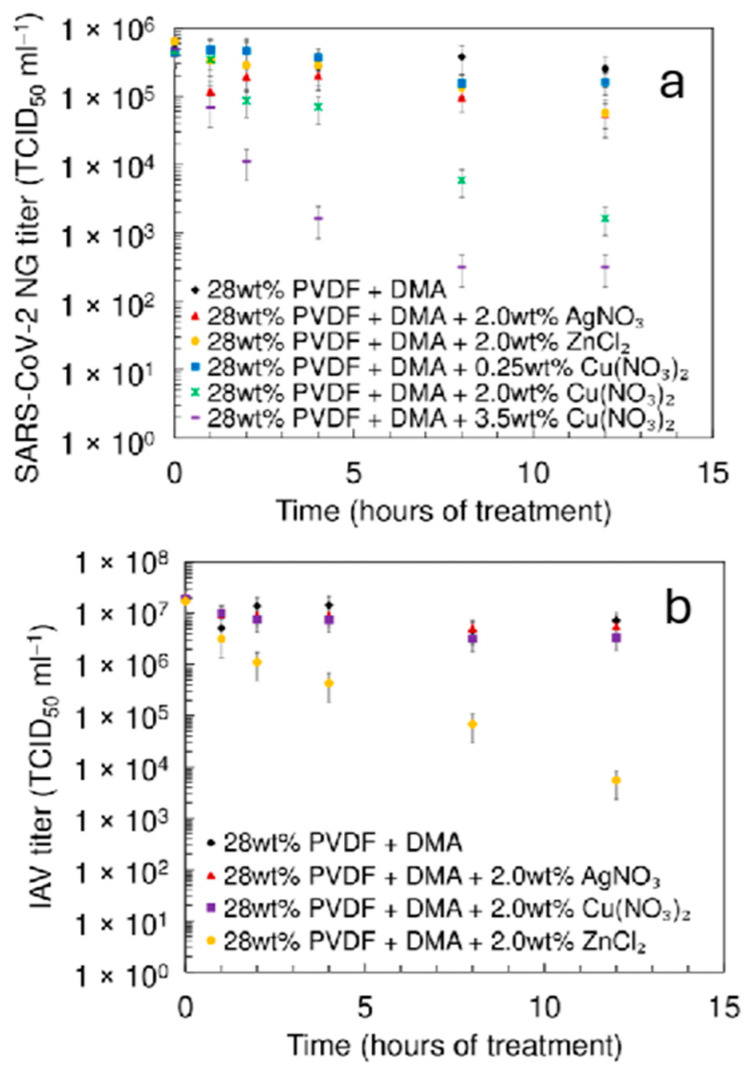
Virudical activity against (**a**) *SARS-CoV-2* and (**b**) human *influenza A virus* H1N1 of electrospun filter materials fabricated using a solution of 28 wt% PVDF + DMA at voltage 9 kV, and with the addition of 2.0 wt% AgNO_3_ at 15 kV, 2.0 wt% ZnCl_2_ at 15 kV, 0.25 wt% Cu(NO_3_)_2_·2.5H_2_O at 17 kV, or 2.0 wt% Cu(NO_3_)_2_·2.5H_2_O at 17 kV, and 3.5 wt% Cu(NO_3_)_2_·2.5H_2_O at 17 kV. Data are represented as means with standard deviations.

Based on the systematic analyses of data, uniform fibrous materials were produced under optimal processing conditions: 60% relative humidity, a solution feed rate of 1 mL h^−1^, a needle-to-collector distance of 9 cm, and an applied voltage of 9 kV for a solution of 28 wt% PVDF + DMA. Selected material was used as a control material to test the virucidal effect of metal nanoparticle materials. For electrospinning the 28 wt% PVDF + DMA solutions with additions of salts (2.0 wt% AgNO_3_, 2.0 wt% ZnCl_2_ or 0.25, 2.0 and 3.5 wt% Cu(NO_3_)_2_·2.5 H_2_O), a voltage of 15 or 17 kV was applied. The virucidal activity of the materials was evaluated against the *SARS-CoV-2* alpha variant and the human *influenza A virus* (*IAV*) A/WSN/1933, with the results presented in Figure 5 [24,26,62].

Experimental results (Figure 5a) show that the virucidal activity of the materials—both without additives and with low concentrations of Cu(NO_3_)_2_·2.5H_2_O (0.25 wt% or 0.75 wt%)—against *SARS-CoV-2* was minimal. After 12 h of contact, the reduction in infectious virus particles (viral titer, Δlog_10_c) was only 0.5 ± 0.05. This indicates that the metal nanoclusters are shielded by a polymer layer [24,26], and the virions cannot contact the active nanoclusters formed during the electrospinning process. It is expected that the higher the overall concentration of the metal nanoparticles in the filter, the higher the concentration of unshielded nanoparticles on the surface of the fibers, and vice versa. The filter materials activated with 2.0 wt% Ag and Zn nanoclusters exhibited somewhat higher activity (Figure 5a). In sharp contrast, the electrospun filter materials created with 2.0 wt% or 3.5 wt% copper salt in the spinning solution showed very high virucidal activity. The reduction in viral titer Δlog*c* was 2.5 ± 0.35 and 3.2 ± 0.30 within 12 h, indicating very high virus-killing efficacy. For the Rouaite nanocluster-activated material, a contact time of just 2 h resulted in a Δlog*c* of 1.7 ± 0.35, demonstrating high virucidal activity. Unmodified membranes, along with those modified with Ag, Zn, and Cu nanoclusters, were tested against the human *influenza A virus* (*IAV*) A/WSN/1933 (H1N1) strain, which had a viral titer of approximately 2.25 ± 1.05 × 10^7^ TCID_50_ mL^−1^. This assessment was conducted following the regulations outlined in the standard document EN 14683:2019 + AC:2019 (ISO 21702:2019), titled “Measurement of the Antiviral Activity on Plastics and Other Non-Porous “Surfaces”. The data are presented in Figure 5b. Membranes modified with Ag nanoclusters (2.0 wt%), as well as with Rouaite nanoclusters (2.0 wt% of Cu(NO_3_)_2_⋅2.5H_2_O), exhibited less pronounced virucidal activity. After 12 h of contact, the reduction in infectious virus particles (Δlog*c*) was approximately 0.5 ± 0.25, indicating low virucidal activity. The moderate activity observed in the Ag and Cu nanocluster-activated materials for the *influenza A virus* can be attributed to the differing hydrophilicities of the nanowire fibers, resulting from the different metals used. The materials with higher porosity (2.0 wt% and 3.5 wt% Cu salt in spinning solution) show more expressed virucidal activity due to higher attainable surface area (more expressed macroporosity, increasing mass transfer into the porous structure of membranes). Surprisingly, the Zn nanocluster-modified membrane exhibited excellent activity, achieving a Δlog*c* 3.0 ± 0.50 within a 12 h test period. The higher hydrophilicity and the more expressed macroporous structure of the given material can explain this. Further studies will be conducted to evaluate the prolonged virucidal activity of all Ag, Zn, and Cu nanocluster-modified membrane materials in the coming months.

## 4. Conclusions

Nanowire poly(vinylidene fluoride) (PVDF) membranes incorporating Cu, Ag, and Zn nanoclusters were fabricated via electrospinning. The membrane structure was tailored by varying the polymer concentration in N,N-dimethylacetamide (DMA), the applied electric field strength, and the concentration of added salts. Hierarchically porous nanofibrous filter materials were produced by electrospinning a 28 wt% PVDF solution in DMA, both with and without the addition of different salts: 2.0 wt% AgNO_3_; 0.25, 2.0, and 3.5 wt% Cu(NO_3_)_2_·2.5H_2_O; or 0.25, 0.75, and 2.0 wt% ZnCl_2_.

X-ray diffraction, scanning electron microscopy combined with energy-dispersive X-ray spectroscopy (SEM–EDX), microwave plasma atomic emission spectroscopy (MP-AES), thermogravimetric analysis (TGA), contact angle measurements, nitrogen sorption, and mercury intrusion porosimetry were used to characterize the structure and chemical composition of the electrospun materials activated with Zn, Cu, and Ag nanoclusters. To evaluate the suitability of these filter materials for use as active layers in multilayer face masks and indoor air filtration systems with virucidal properties, we tested their particle filtration efficiency and antiviral activity against human *influenza A virus* (*IAV*) A/WSN/1933 (H1N1) and the *SARS-CoV-2* alpha variant.

All filter materials prepared with salts in the spinning solution demonstrated very high particle filtration efficiency (≥98.0%). Additionally, the hydrophobicity of the membranes was evaluated through contact angle measurements, revealing that all membrane surfaces exhibit a high degree of hydrophobicity; however, the addition of metal nanoclusters makes fiber materials slightly more hydrophilic.

The best virucidal activity against the *SARS-CoV-2* alpha variant was observed if material electrospun from a spinning solution containing Cu(NO_3_)_2_⋅2.5H_2_O was used. This material showed a reduction in infectious virions by three orders of magnitude after 12 h of contact. In contrast, materials containing Ag and Zn nanoclusters reduced infectious virions by only ~8 and ~11 times, respectively, over the same duration. Additionally, Cu nanoclusters’ concentration in PVDF membranes was determined, and their influence on the virucidal activity against *SARS-CoV-2* alpha variant was established. The virucidal activity increased with the increase in the concentration of Cu nanoclusters in the fibers under study. The most active material against the human *influenza A virus* A/WSN/1933 (H1N1) was a fibrous nanowire polymer activated with Zn nanoclusters. Thus, it can be concluded based on the data collected and analyzed that materials with Cu nanoclusters can be used with high efficiency to passivate and kill *SARS-CoV-2* alpha variant virions, and Zn nanocluster-modified activated porous membranes can be used for killing human *influenza A virus* A7WSN/1933 (H1N1) virions.

## Figures and Tables

**Table 1:** Nitrogen sorption and mercury intrusion porosimetry analysis results are presented for electrospun filter materials made from a 28 wt% PVDF + DMA solution, both with and without the addition of x wt% AgNO_3_, ZnCl_2_, or Cu(NO_3_)_2_·2.5H_2_O, as specified in the table.

Solution	Nitrogen Sorption		Mercury Intrusion		
	*S*_BET_ (m^2^ g^−1^)	*V*_tot_ (cm^3^ g^−1^)	*S*_Hg_ (m^2^ g^−1^)	*V*_Hg_ (cm^3^ g^−1^)	Porosity (%)
28 wt% PVDF + DMA	9.6	0.015	23.5	3.9	28
28 wt% PVDF + DMA + 2.0 wt% AgNO_3_	4.5	0.0075	22.5	4.7	26
28 wt% PVDF + DMA + 2.0 wt% ZnCl_2_	4.4	0.0067	34.0	4.1	27
28 wt% PVDF + DMA + 0.25 wt% Cu(NO_3_)_2_·2.5H_2_O	3.6	0.0057	10.5	4.3	26
28 wt% PVDF + DMA + 0.75 wt% Cu(NO_3_)_2_·2.5H_2_O	4.3	0.0060	7.5	3.8	29
28 wt% PVDF + DMA + 2.0 wt% Cu(NO_3_)_2_·2.5H_2_O	4.3	0.0057	8.0	3.9	27
28 wt% PVDF + DMA + 3.5 wt% Cu(NO_3_)_2_·2.5H_2_O	2.5	0.0035	11.0	3.0	32

**Table 2:** Particle filtration efficiency (*E*_eff_) and pressure drop were evaluated for electrospun filter materials produced from a 28 wt% PVDF + DMA solution, both without and with the addition of x wt% AgNO_3_, ZnCl_2_, or Cu(NO_3_)_2_·2.5H_2_O, as specified in the Table.

Solution	Filter Thickness (μm)	*E*_eff_, 100 nm (%)	*E*_eff_*,* 300 nm (%)	*E*_eff_, 3000 nm (%)	Pressure Drop (Pa cm^−2^)
28 wt% PVDF + DMA	55 ± 5	65 ± 2	76 ± 0.1	99.5 ± 0.1	53 ± 6
	85 ± 5	84 ± 4	92 ± 2.0	99.9 ± 0.1	51 ± 5
	130 ± 8	96 ± 1	99 ± 0.2	99.9 ± 0.0	144 ± 15
28 wt% PVDF + DMA + 2.0 wt% AgNO_3_	100 ± 10	100 ± 0.0	99 ± 0.2	99.9 ± 0.1	685 ± 60
28 wt% PVDF + DMA + 0.25 wt% ZnCl_2_	40 ± 5	99.7 ± 0.1	98 ± 0.3	99.9 ± 0.1	105 ± 10
	75 ± 5	100 ± 0.0	99.8 ± 0.1	99.9 ± 0.1	205 ± 15
	160 ± 10	100 ± 0.0	99.8 ± 0.1	99.9 ± 0.1	335 ± 15
28 wt% PVDF + DMA + 0.75 wt% ZnCl_2_	70 ± 5	99.9 ± 0.1	98.9 ± 0.2	99.7 ± 0.1	115 ± 15
	125 ± 10	100 ± 0.0	99.3 ± 0.1	99.9 ± 0.1	335 ± 15
28 wt% PVDF + DMA + 2.0 wt% ZnCl_2_	30 ± 5	98.9 ± 0.1	99 ± 0.2	99.9 ± 0.1	62 ± 9.5
28 wt% PVDF + DMA + 0.75 wt% Cu(NO_3_)_2_·2.5H_2_O	25 ± 3	100 ± 0.0	100 ± 0.0	100 ± 0.0	130 ± 2
	100 ± 10	100 ± 0.0	100 ± 0.0	100 ± 0.0	455 ± 10
	200 ± 20	100 ± 0.0	100 ± 0.0	100 ± 0.0	820 ± 50
28 wt% PVDF + DMA + 2.0 wt% Cu(NO_3_)_2_·2.5H_2_O	35 ± 4	98.5 ± 0.5	99.7 ± 0.2	100 ± 0.3	105 ± 2
	90 ± 8	100 ± 0.0	100 ± 0.1	100 ± 0.0	390 ± 85
	145 ± 10	99.9 ± 0.1	99.9 ± 0.1	99.9 ± 0.1	695 ± 75
28 wt% PVDF + DMA + 3.5 wt% Cu(NO_3_)_2_·2.5H_2_O	30 ± 5	95.5 ± 4.0	100 ± 0.0	100 ± 0.0	85 ± 15
	110 ± 15	100 ± 0.0	100 ± 0.0	100 ± 0.0	145 ± 15
	200 ± 20	100 ± 0.0	100 ± 0.0	100 ± 0.0	400 ± 95

## Data Availability

All data are included within the article; additional inquiries can be addressed to the corresponding author.

## Supplementary Materials

The following supporting information can be downloaded at: https://www.mdpi.com/article/10.3390/pharmaceutics17070887/s1, Figure S1. Scheme of electrospinning system with syringe pump, high voltage source, rail, rolling collector and climate control unit. Figure S2. XRD patterns for electrospun filter materials fabricated using a solution of 28 wt% PVDF + DMA at voltage of 9 kV and with the addition of 2.0 wt% AgNO_3_ at 15 kV and 2.0 wt% ZnCl_2_ at 15 kV and 0.25 wt% Cu(NO_3_)_2_·2.5H_2_O at 17 kV and 3.5 wt% Cu(NO_3_)_2_·2.5H_2_O at 17 kV (noted in the figure). Rouaite, *β*-PVDF and *γ*-PVDF form a crystal structure, and their reflections are provided for comparison. Table S1. Electrospun filter materials preparation conditions and average fiber size (*d*_av_) dependency of applied voltage and salt concentration is a solution. Needle to collector distance was constant at 9 cm. Table S2. The concentration of metals in electrospun filter materials established by MP–AES compared to theoretical values.

## References

[1] Wibisono Y., Fadila C.R., Saiful S., Bilad M.R. (2020). Facile Approaches of Polymeric Face Masks Reuse and Reinforcements for Micro-Aerosol Droplets and Viruses Filtration: A Review. Polymers.

[2] Cook T.M. (2020). Personal Protective Equipment during the Coronavirus Disease (COVID) 2019 Pandemic—A Narrative Review. Anaesthesia.

[3] Prather K.A., Wang C.C., Schooley R.T. (2020). Reducing Transmission of SARS-CoV-2. Science.

[4] Teo J.Y., Kng J., Periaswamy B., Liu S., Lim P., Lee C.E., Tan B.H., Loh X.J., Ni X., Tiang D. (2021). Exploring Reusability of Disposable Face Masks: Effects of Disinfection Methods on Filtration Efficiency, Breathability, and Fluid Resistance. Glob. Chall..

[5] Rubio-Romero J.C., Pardo-Ferreira M.d.C., Torrecilla-García J.A., Calero-Castro S. (2020). Disposable Masks: Disinfection and Sterilization for Reuse, and Non-Certified Manufacturing, in the Face of Shortages during the COVID-19 Pandemic. Saf. Sci..

[6] Li A.S.H., Sathishkumar P., Selahuddeen M.L., Mahmood W.M.A.W., Abidin M.H.Z., Wahab R.A., Huri M.A.M., Abdullah F. (2022). Adverse Environmental Effects of Disposable Face Masks Due to the Excess Usage. Environ. Pollut..

[7] (2017). High Efficiency Filters and Filter Media for Removing Particles from Air—Part 1: Classification, Performance, Testing and Marking.

[8] Zhou Z., You T., Pan Z., Wang D., Wang H., Wang L., Xu G., Liang Y., Hu J., Tang M. (2024). Trichome-Like Biomimetic Air Filters via Templated Silicone Nanofilaments. Adv. Mater..

[9] Zhou Z., You T., Wang D., Pan Z., Xu G., Liang Y., Tang M. (2024). Conformal Build-Up of Functionalized Air Filters with Improved Air Cleaning and Bioprotective Traps. Adv. Funct. Mater..

[10] Jazie A.A., Albaaji A.J., Abed S.A. (2021). A Review on Recent Trends of Antiviral Nanoparticles and Airborne Filters: Special Insight on COVID-19 Virus. Air Qual. Atmos. Health.

[11] Ge Z., Yang L., Xia J., Fu X., Zhang Y. (2020). Possible Aerosol Transmission of COVID-19 and Special Precautions in Dentistry. J. Zhejiang Univ. Sci. B.

[12] Mamun A., Blachowicz T., Sabantina L. (2021). Electrospun Nanofiber Mats for Filtering Applications—Technology, Structure and Materials. Polymers.

[13] Lee E.C., Wada N.I., Grabowski M.K., Gurley E.S., Lessler J. (2020). The Engines of SARS-CoV-2 Spread. Science.

[14] Babaahmadi V., Amid H., Naeimirad M., Ramakrishna S. (2021). Biodegradable and Multifunctional Surgical Face Masks: A Brief Review on Demands during COVID-19 Pandemic, Recent Developments, and Future Perspectives. Sci. Total Environ..

[15] Wang C.C., Prather K.A., Sznitman J., Jimenez J.L., Lakdawala S.S., Tufekci Z., Marr L.C. (2021). Airborne Transmission of Respiratory Viruses. Science.

[16] Du H., Huang S., Wang J. (2022). Environmental Risks of Polymer Materials from Disposable Face Masks Linked to the COVID-19 Pandemic. Sci. Total Environ..

[17] Türkmen B.A. (2022). Life Cycle Environmental Impacts of Disposable Medical Masks. Environ. Sci. Pollut. Res..

[18] Liu R., Mabury S.A. (2021). Single-Use Face Masks as a Potential Source of Synthetic Antioxidants to the Environment. Environ. Sci. Technol. Lett..

[19] Prata J.C., Silva A.L.P., Walker T.R., Duarte A.C., Rocha-Santos T. (2020). COVID-19 Pandemic Repercussions on the Use and Management of Plastics. Environ. Sci. Technol..

[20] Chang S.-Y., Huang K.-Y., Chao T.-L., Kao H.-C., Pang Y.-H., Lu L., Chiu C.-L., Huang H.-C., Cheng T.-J.R., Fang J.-M. (2021). Nanoparticle Composite TPNT1 Is Effective against SARS-CoV-2 and Influenza Viruses. Sci. Rep..

[21] Kim S., Chung J., Lee S.H., Yoon J.H., Kweon D.-H., Chung W.-J. (2021). Tannic Acid-Functionalized HEPA Filter Materials for Influenza Virus Capture. Sci. Rep..

[22] Mallakpour S., Azadi E., Hussain C.M. (2022). Fabrication of Air Filters with Advanced Filtration Performance for Removal of Viral Aerosols and Control the Spread of COVID-19. Adv. Colloid Interface Sci..

[23] Lee J., Bae J., Youn D.-Y., Ahn J., Hwang W.-T., Bae H., Bae P.K., Kim I.-D. (2022). Violacein-Embedded Nanofiber Filters with Antiviral and Antibacterial Activities. Chem. Eng. J..

[24] Thomberg T., Bulgarin H., Lust A., Nerut J., Koppel M., Romann T., Palm R., Månsson M., March N.M.F., Junninen H. (2023). The Anti SARS-CoV-2 Activity of Nanofibrous Filter Materials Activated with Metal Clusters. Atmos. Environ. X.

[25] Thomberg T., Ramah P., Lust A., Nerut J., Koppel M., Romann T., Palm R., Månsson M., March N.M.F., Junninen H. (2022). Preparation of Nanofibrous Materials Activated with Metal Clusters for Active and Long-Lasting Air Filters. Sep. Purif. Technol..

[26] Bulgarin H., Thomberg T., Lust A., Nerut J., Koppel M., Romann T., Palm R., Månsson M., Vana M., Junninen H. (2024). Enhanced and Copper Concentration Dependent Virucidal Effect against SARS-CoV-2 of Electrospun Poly(Vinylidene Difluoride) Filter Materials. iScience.

[27] Lu T., Cui J., Qu Q., Wang Y., Zhang J., Xiong R., Ma W., Huang C. (2021). Multistructured Electrospun Nanofibers for Air Filtration: A Review. ACS Appl. Mater. Interfaces.

[28] Senthil R., Sumathi V., Tamilselvi A., Kavukcu S.B., Aruni A.W. (2022). Functionalized Electrospun Nanofibers for High Efficiency Removal of Particulate Matter. Sci. Rep..

[29] Karabulut F.N.H., Höfler G., Ashok Chand N., Beckermann G.W. (2021). Electrospun Nanofibre Filtration Media to Protect against Biological or Nonbiological Airborne Particles. Polymers.

[30] Tõnurist K., Jänes A., Thomberg T., Kurig H., Lust E. (2009). Influence of Mesoporous Separator Properties on the Parameters of Electrical Double-Layer Capacitor Single Cells. J. Electrochem. Soc..

[31] Tõnurist K., Thomberg T., Jänes A., Lust E. (2013). Specific Performance of Supercapacitors at Lower Temperatures Based on Different Separator Materials. J. Electrochem. Soc..

[32] Tõnurist K., Thomberg T., Jänes A., Kink I., Lust E. (2012). Specific Performance of Electrical Double Layer Capacitors Based on Different Separator Materials in Room Temperature Ionic Liquid. Electrochem. Commun..

[33] Tõnurist K., Vaas I., Thomberg T., Jänes A., Kurig H., Romann T., Lust E. (2014). Application of Multistep Electrospinning Method for Preparation of Electrical Double-Layer Capacitor Half-Cells. Electrochim. Acta.

[34] Tõnurist K. (2013). Influence of Electrospun Separator Materials Properties on Electrochemical Performance of Electrical Double-Layer Capacitors. Ph.D. Thesis.

[35] Ramakrishna S. (2005). An Introduction to Electrospinning and Nanofibers.

[36] Kubo A.-L., Rausalu K., Savest N., Žusinaite E., Vasiliev G., Viirsalu M., Plamus T., Krumme A., Merits A., Bondarenko O. (2022). Antibacterial and Antiviral Effects of Ag, Cu and Zn Metals, Respective Nanoparticles and Filter Materials Thereof against Coronavirus SARS-CoV-2 and Influenza A Virus. Pharmaceutics.

[37] Laidmäe I., Meos A., Kjærvik I.A., Ingebrigtsen S.G., Škalko-Basnet N., Kirsimäe K., Romann T., Joost U., Kisand V., Kogermann K. (2021). Electrospun Amphiphilic Nanofibers as Templates for In Situ Preparation of Chloramphenicol-Loaded Liposomes. Pharmaceutics.

[38] Ivask A., Juganson K., Bondarenko O., Mortimer M., Aruoja V., Kasemets K., Blinova I., Heinlaan M., Slaveykova V., Kahru A. (2014). Mechanisms of Toxic Action of Ag, ZnO and CuO Nanoparticles to Selected Ecotoxicological Test Organisms and Mammalian Cells in Vitro: A Comparative Review. Nanotoxicology.

[39] Jeremiah S.S., Miyakawa K., Morita T., Yamaoka Y., Ryo A. (2020). Potent Antiviral Effect of Silver Nanoparticles on SARS-CoV-2. Biochem. Biophys. Res. Commun..

[40] Galdiero S., Falanga A., Vitiello M., Cantisani M., Marra V., Galdiero M. (2011). Silver Nanoparticles as Potential Antiviral Agents. Molecules.

[41] Qamar H., Rehman S., Chauhan D.K., Tiwari A.K., Upmanyu V. (2020). Green Synthesis, Characterization and Antimicrobial Activity of Copper Oxide Nanomaterial Derived from Momordica Charantia. Int. J. Nanomed..

[42] Aallaei M., Molaakbari E., Mostafavi P., Salarizadeh N., Maleksah R.E., Afzali D. (2022). Investigation of Cu Metal Nanoparticles with Different Morphologies to Inhibit SARS-CoV-2 Main Protease and Spike Glycoprotein Using Molecular Docking and Dynamics Simulation. J. Mol. Struct..

[43] Govind V., Bharadwaj S., Sai Ganesh M.R., Vishnu J., Shankar K.V., Shankar B., Rajesh R. (2021). Antiviral Properties of Copper and Its Alloys to Inactivate Covid-19 Virus: A Review. Biometals.

[44] Rani I., Goyal A., Bhatnagar M., Manhas S., Goel P., Pal A., Prasad R. (2021). Potential Molecular Mechanisms of Zinc- and Copper-Mediated Antiviral Activity on COVID-19. Nutr. Res..

[45] Bahrami A., Arabestani M.R., Taheri M., Farmany A., Norozzadeh F., Hosseini S.M., Nozari H., Nouri F. (2022). Exploring the Role of Heavy Metals and Their Derivatives on the Pathophysiology of COVID-19. Biol. Trace Elem. Res..

[46] Maduray K., Parboosing R. (2021). Metal Nanoparticles: A Promising Treatment for Viral and Arboviral Infections. Biol. Trace Elem. Res..

[47] Toledo G.G.D., Toledo V.H., Lanfredi A.J.C., Escote M., Champi A., Silva M.C.C.D., Nantes-Cardoso I.L. (2020). Promising Nanostructured Materials against Enveloped Virus. An. Acad. Bras. Ciênc..

[48] de Jesus Silva A.J., Contreras M.M., Nascimento C.R., da Costa M.F. (2020). Kinetics of Thermal Degradation and Lifetime Study of Poly(Vinylidene Fluoride) (PVDF) Subjected to Bioethanol Fuel Accelerated Aging. Heliyon.

[49] Balaram V. (2020). Microwave Plasma Atomic Emission Spectrometry (MP-AES) and Its Applications—A Critical Review. Microchem. J..

[50] Hotaling N.A., Bharti K., Kriel H., Simon C.G. (2015). DiameterJ: A Validated Open Source Nanofiber Diameter Measurement Tool. Biomaterials.

[51] (2019). Medical Face Masks—Requirements and Test Methods.

[52] Thommes M., Kaneko K., Neimark A.V., Olivier J.P., Rodriguez-Reinoso F., Rouquerol J., Sing K.S.W. (2015). Physisorption of Gases, with Special Reference to the Evaluation of Surface Area and Pore Size Distribution (IUPAC Technical Report). Pure Appl. Chem..

[53] Rouquerol J., Rouquerol F., Sing K.S.W. (1998). Adsorption by Powders and Porous Solids: Principles, Methodology and Applications.

[54] Webb P.A., Orr C., Camp R.W., Olivier J.P., Yunes Y.S. (1997). Analytical Methods in Fine Particle Technology.

[55] Plötze M., Niemz P. (2011). Porosity and Pore Size Distribution of Different Wood Types as Determined by Mercury Intrusion Porosimetry. Eur. J. Wood Prod..

[56] Wang S., Yang P., Dai D., Xue K., Li D. (2020). A Study on Micro-Pore Characteristics of Clay Due to Freeze-Thaw and Compression by Mercury Intrusion Porosimetry. Front. Earth Sci..

[57] Cseri T., Békássy S., Kenessey G., Liptay G., Figueras F. (1996). Characterization of Metal Nitrates and Clay Supported Metal Nitrates by Thermal Analysis. Thermochim. Acta.

[58] Holder C.F., Schaak R.E. (2019). Tutorial on Powder X-Ray Diffraction for Characterizing Nanoscale Materials. ACS Nano.

[59] Cai X., Lei T., Sun D., Lin L. (2017). A Critical Analysis of the α, β and γ Phases in Poly(Vinylidene Fluoride) Using FTIR. RSC Adv..

[60] Wu T., Zhou B., Zhu T., Shi J., Xu Z., Hu C., Wang J. (2015). Facile and Low-Cost Approach towards a PVDF Ultrafiltration Membrane with Enhanced Hydrophilicity and Antifouling Performance via Graphene Oxide/Water-Bath Coagulation. RSC Adv..

[61] (2019). Measurement of Antiviral Activity on Plastics and Other Non-Porous Surfaces.

[62] Rihn S.J., Merits A., Bakshi S., Turnbull M.L., Wickenhagen A., Alexander A.J.T., Baillie C., Brennan B., Brown F., Brunker K. (2021). A Plasmid DNA-Launched SARS-CoV-2 Reverse Genetics System and Coronavirus Toolkit for COVID-19 Research. PLOS Biol..

[63] Wang W., Zhang S., Srisombat L.O., Lee T.R., Advincula R.C. (2011). Gold-Nanoparticle- and Gold-Nanoshell-Induced Polymorphism in Poly(Vinylidene Fluoride). Macromol. Mater. Eng..

[64] Mandal D., Kim K.J., Lee J.S. (2012). Simple Synthesis of Palladium Nanoparticles, β-Phase Formation, and the Control of Chain and Dipole Orientations in Palladium-Doped Poly(Vinylidene Fluoride) Thin Films. Langmuir.

[65] Kim H.B., Lee W.J., Choi S.C., Lee K.B., Lee M.-H. (2021). Filter Quality Factors of Fibrous Filters with Different Fiber Diameter. Aerosol Sci. Technol..

[66] Zhao X., Wang S., Yin X., Yu J., Ding B. (2016). Slip-Effect Functional Air Filter for Efficient Purification of PM2.5. Sci. Rep..

[67] Delumeau L.-V., Asgarimoghaddam H., Alkie T., Jones A.J.B., Lum S., Mistry K., Aucoin M.G., DeWitte-Orr S., Musselman K.P. (2021). Effectiveness of Antiviral Metal and Metal Oxide Thin-Film Coatings against Human Coronavirus 229E. APL Mater..

[68] Richardson H.W. (2000). Copper Compounds. Ullmann’s Encyclopedia of Industrial Chemistry.

