# Supplementary material for: Synthesis and Antiviral Activity of Nanowire Polymers Activated with Ag, Zn, and Cu Nanoclusters
Source: Pharmaceutics. 2025 Jul 6;17(7):887. doi: 10.3390/pharmaceutics17070887 (PMC12298448; doi:10.3390/pharmaceutics17070887)
Supplement: Supplementary file 1 [file pharmaceutics-17-00887-s001.zip › pharmaceutics-3686927-supplementary.pdf]

Article

Supplementary Materials: Synthesis and Antiviral Activity of Nanowire Polymers Activated with Ag, Zn, and Cu Nanoclusters

T. Thomberg, H. Bulgarin, A. Lust, J. Nerut, T. Romann, E. Lust

Contents of the supporting information:

Fig. S1. Scheme of electrospinning system.

Fig. S2. XRD patterns for electrospun filter materials.

Table S1. Electrospun filter materials preparation conditions.

Table S2. Metal nanocluster content in electrospun filter materials.

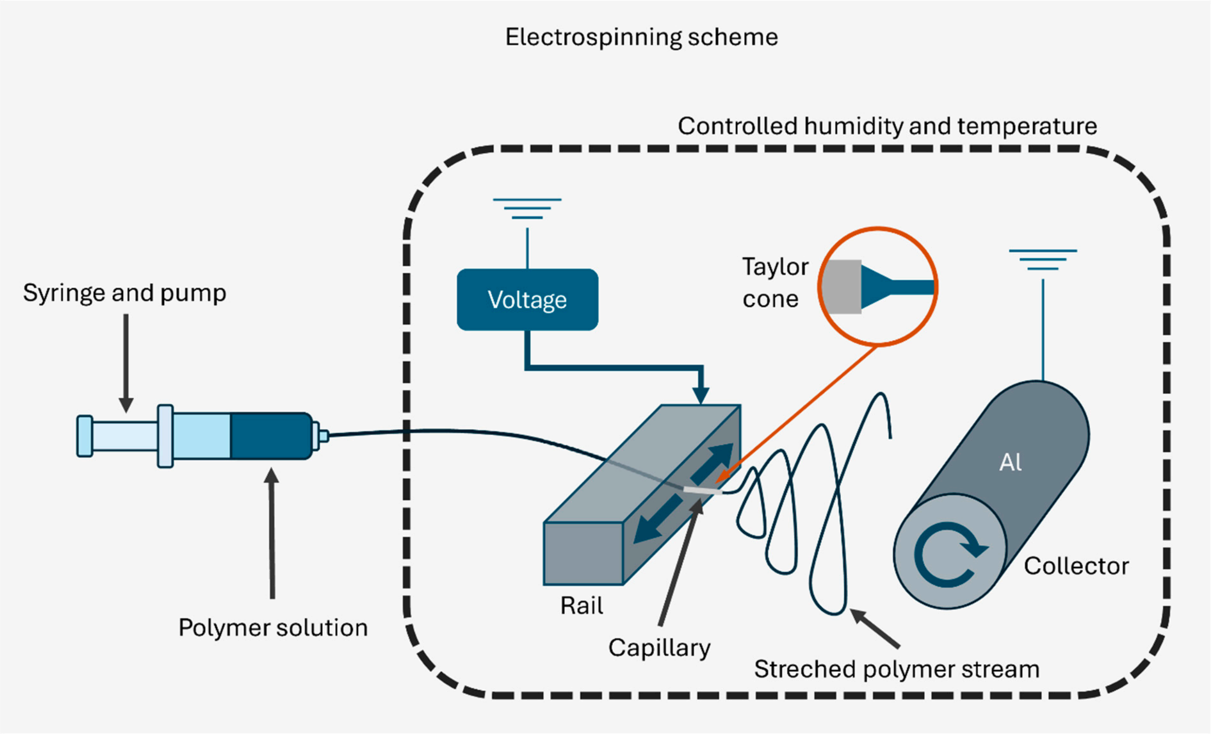

Fig. S1. Scheme of electrospinning system with syringe pump, high voltage source, rail, rolling collector and climate control unit.

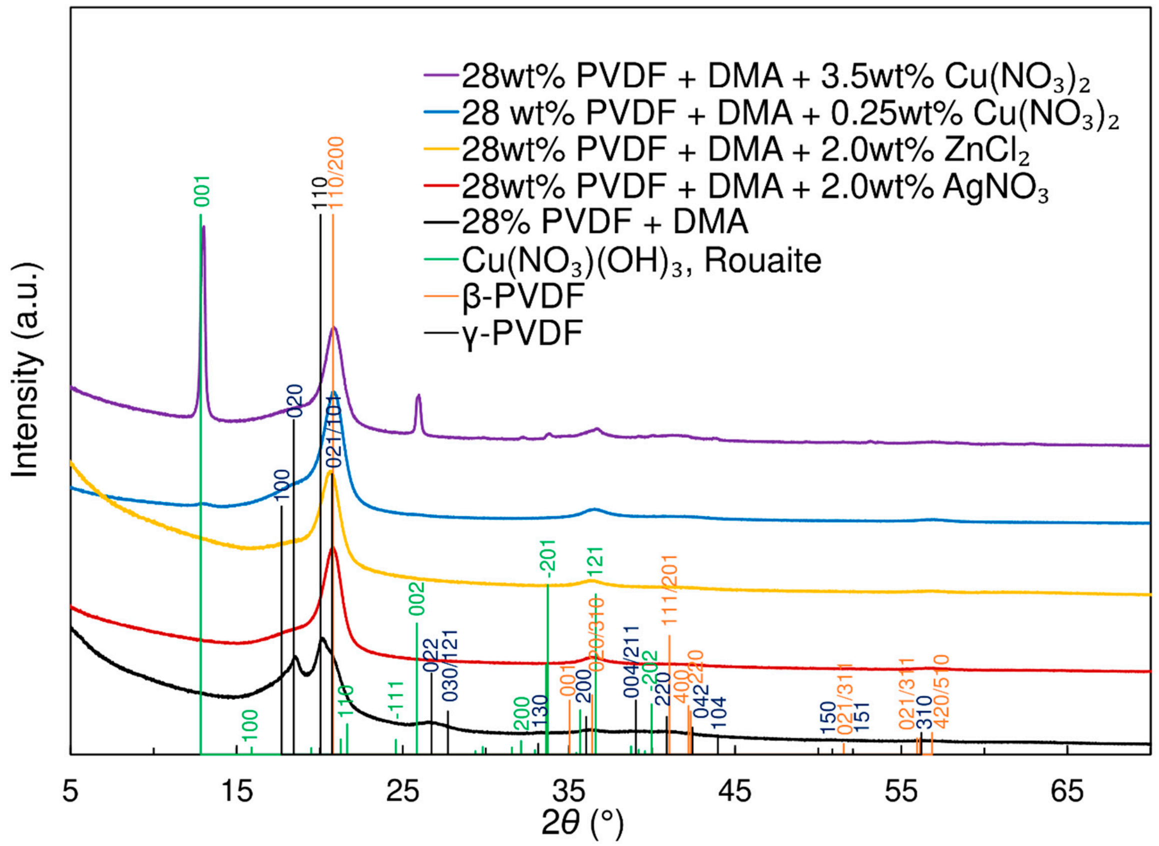

Fig. S2. XRD patterns for electrospun filter materials fabricated using a solution of 28 wt% PVDF + DMA at voltage of 9 kV and with the addition of 2.0 wt% $AgNO_3$ at 15 kV and 2.0 wt% $ZnCl_2$ at 15 kV and 0.25 wt% $Cu(NO_3)_2 \cdot 2.5H_2O$ at 17 kV and 3.5 wt% $Cu(NO_3)_2 \cdot 2.5H_2O$ at 17 kV (noted in the figure). Rouaite, β–PVDF and γ–PVDF form a crystal structure, and their reflections are provided for comparison.

Table S1. Electrospun filter materials preparation conditions and average fiber size (d_{av}) dependency of applied voltage and salt concentration is a solution. Needle to collector distance was constant at 9 cm.

Solution	Voltage (kV)	d_{av} (nm)
28wt% PVDF + DMA	9	490 ± 350
	13	610 ± 380
28wt% PVDF + DMA + 0.25 wt% $AgNO_3$	11	490 ± 240
	13	400 ± 200
	15	510 ± 180
	17	460 ± 190
	19	470 ± 190
28wt% PVDF + DMA + 0.75 wt% $AgNO_3$	11	470 ± 200
	13	540 ± 250
	15	490 ± 190
	17	500 ± 180
	19	530 ± 160
28wt% PVDF + DMA + 2.0 wt% $AgNO_3$	11	N/A
	13	N/A
	15	560 ± 200
	17	N/A
	19	N/A
28wt% PVDF + DMA + 0.25 wt% $ZnCl_2$	9	960 ± 230
	11	770 ± 230
	13	680 ± 320
	15	710 ± 200
	17	670 ± 170
28wt% PVDF + DMA + 0.75 wt% $ZnCl_2$	9	640 ± 220
	11	630 ± 220
	13	540 ± 200
	15	590 ± 240
	17	650 ± 490
28wt% PVDF + DMA + 2.0 wt% $ZnCl_2$	9	450 ± 120
	11	640 ± 140
	13	530 ± 220
	15	430 ± 110
	17	440 ± 100
28 wt% PVDF + DMA + 0.25 wt% $Cu(NO_3)_2 \cdot 2.5H_2O$	11	390 ± 100
	13	420 ± 90
	15	470 ± 90
	17	420 ± 80
	19	410 ± 100
28 wt% PVDF + DMA + 0.75 wt% $Cu(NO_3)_2 \cdot 2.5H_2O$	13	450 ± 120
	15	500 ± 100
	17	470 ± 120
	19	460 ± 110
	21	410 ± 80
28 wt% PVDF + DMA + 2.0 wt% $Cu(NO_3)_2 \cdot 2.5H_2O$	13	530 ± 220
	15	480 ± 160

	17	500 ± 100
	19	520 ± 100
	21	500 ± 100
28 wt% PVDF + DMA + 3.5 wt% $Cu(NO_3)_2·2.5H_2O$	13	560 ± 170
	15	670 ± 260
	17	520 ± 90
	19	600 ± 100
	21	550 ± 90

Table S2. The concentration of metals in electrospun filter materials established by MP−AES compared to theoretical values.

Solution	Theoretical (wt%)	MP−AES (wt%)
28 wt% PVDF + DMA + 2.0 wt% $AgNO_3$	4.34	1.37 ± N/A
28 wt% PVDF + DMA + 0.25 wt% $ZnCl_2$	0.426	0.44 ± N/A
28 wt% PVDF + DMA + 0.75 wt% $ZnCl_2$	1.27	1.23 ± N/A
28 wt% PVDF + DMA + 2.0 wt% $ZnCl_2$	3.31	3.33 ± N/A
28 wt% PVDF + DMA + 0.25 wt% $Cu(NO_3)_2·2.5H_2O$	0.242	0.242 ± 0.001
28 wt% PVDF + DMA + 0.75 wt% $Cu(NO_3)_2·2.5H_2O$	0.713	0.695 ± 0.002
28 wt% PVDF + DMA + 2.0 wt% $Cu(NO_3)_2·2.5H_2O$	1.82	1.750 ± 0.006
28 wt% PVDF + DMA + 3.5 wt% $Cu(NO_3)_2·2.5H_2O$	3.03	2.916 ± 0.02
